# Introduction of day-case robotic liver surgery: a case series from a tertiary hepatobiliary and pancreatic centre

**DOI:** 10.1007/s00464-024-10913-9

**Published:** 2024-06-14

**Authors:** Kai Tai Derek Yeung, Rajendran Vellaisamy, Aasim Hussain, Olivia Mingo, Ravishankar Raobaikady, David Nicol, Shahnawaz Rasheed, Paris Tekkis, David Cunningham, Long R. Jiao

**Affiliations:** 1 Department of Surgery and Oncology, The Royal Marsden Hospital, 203 Fulham Road, London, SW3 6JJ UK; 2 Imperial College London, Exhibition Road, South Kensington, London, SW7 2BU UK; 3 Department of Academic Surgery, The Royal Marsden Hospital, Imperial College London, 203 Fulham Road, London, SW3 6JJ UK

**Keywords:** Robotic liver resection, Day case surgery, Liver resection, Colorecal liver metastasis

## Abstract

**Background:**

Liver surgery is associated with a significant hospital stay regardless the type of liver resection. A large incision is essential for open liver surgery which is a major factor in the course of the patient’s recovery. For patients with small parenchyma liver lesions requiring surgical resection, robotic surgery potentially offers the opportunity to transform the patient’s post-operative course. A day-case robotic liver resection pathway was formulated and implemented at our institution when patients were planned for discharge within 24 h of admission for liver surgery.

**Methods:**

Single surgeon case series of cases performed at a tertiary hepatobiliary and pancreatic centre between September 2022 and November 2023. The inclusion criteria were non-anatomical wedge resections, < 2 anatomical segmental resections, left lateral hepatectomy and minimally invasive surgery.

**Results:**

This is the first series of robotic day-case minor liver resection in the United Kingdom. 20 patients were included in this case series. The mean operative time was 86.6 ± 30.9 min and mean console time was 58.6 ± 24.5 min. Thirteen patients (65%) were discharged within 24 h of surgery. The main cause of hospitalisation beyond 24 h was inadequate pain relief. There were no Clavien–Dindo grade III or above complications, no 30-day readmission and 90-day mortalities.

**Conclusion:**

This case series demonstrates that robotic day-case liver resection is safe and feasible. Robust follow-up pathways must be in place to allow for the safe implementation of this approach, to monitor for any complications and to allow intervention as required in a timely manner.

Liver surgery is associated with a significant hospital stay regardless the type of liver resection. A large subcostal “J” shaped or roof top incision is essential for open liver surgery which is a major factor in the course of the patient’s recovery. Minimally invasive surgery (MIS) can reduce the problems associated with such incisions and its benefits are now well recognised [1] including reduced post-operative pain, shorter return to function and reduced length of hospital stay [1] as well as increased cost-effectiveness. Furthermore, the current evidence has clearly shown the benefit of MIS on major abdominal operations with the reduced risks of perioperative complications including reduced blood loss, hospital-acquired infections, reduced wound infections, thromboembolic and cardiopulmonary events [2–5]. An earlier return to home allows patients to recover in familiar surroundings and return to usual activities of daily living. Robotic surgery allows for the benefits of MIS but in addition it offers endowristed instruments for access to the superoposterior parts of liver where it is inaccessible to laparoscopic instruments and full mobilisation of liver which is again difficult to achieve with inherent limitations of rigid laparoscopic tools. Furthermore, it improves surgeon’s ergonomics and dexterity with superior precision and vision. However, robotic surgery is also associated with increased cost, a significant learning curve, requires a bedside robotic assistant and theatre team trained in the utilisation of the surgical robotic systems.

For patients with small parenchyma liver lesions requiring surgical resection, robotic surgery potentially offers the opportunity to transform the patient’s post-operative course. The procedure is followed by a short overnight stay as a day-case on an enhanced recovery pathway. This has the potential to free up hospital bed spaces and to reduce costs. For this to take place safely, careful pre-operative patient assessment and selection are key to ensuring the appropriate patients are selected. Robust follow-up and protocols should also be in place to monitor for any complications and to allow intervention as required in a timely manner.

Robotic liver resection is safe with good outcomes [6] when performed for common hepatic oncological indications such as colorectal liver metastasis [7, 8], cholangiocarcinoma [9], hepatocellular carcinoma [10] and gallbladder fossa resections [11]. In particular, robotic minor liver resections are safe and lead to shorter lengths of stay, with comparable outcomes to open surgery [12, 13]. Same-day discharge following robotic surgery has been utilised and shown to be safe and efficient by other surgical specialities such as urology [14, 15].

On this basis, a novel day-case robotic liver resection pathway was formulated and implemented at our institution when patients were planned for discharge within 24 h of admission for liver surgery. The initial experience of the consecutive cases are reported here.

## Methods

### Selection criteria and pathway protocol

#### Patient selection

All patients underwent computerised tomography (CT) of chest, abdomen and pelvis ± magnetic resonance imaging (MRI) and positron emission tomography (PET/CT) as part of pre-operative staging investigations and were discussed at a weekly dedicated liver multidisciplinary team (MDT) meeting. Patients were deemed suitable for this pathway if the resection involved one to two peripheral non-anatomical liver resections; a European Cooperative Oncology Group (ECOG) performance status of zero or one, as well as an American Society of Anaesthesiologist (ASA) grade of one or two. Exceptions may be made on a case-by-case basis following a thorough consultant surgeon evaluation followed by consultant-led anaesthetic assessment. All procedures were performed by the single surgeon (LRJ) who has performed over 300 robotic liver resections consisting of 70% of liver resection cases by the senior surgeon (LRJ) with 40% being major formal left and right hepatectomy. Currently, the centre volume is 150 liver resections annually, shared between two HPB surgeons.

Criteria for day-case liver resections

- Non anatomical wedge resections
-  < 2 anatomical segmental resection
- Left lateral hepatectomy
- Minimally Invasive Surgery

#### Surgical technique

Following general anaesthesia, pneumoperitoneum is achieved via infra-umbilical open Hassan technique. Following diagnostic laparoscopy to ensure there are no obstructing adhesions or unexpected findings, a further four 8 mm robotic ports are placed under direct vision and docked with Da Vinci Xi Robotic System. An intracorporeal robotic ultrasound scan is utilised if necessary. Liver parenchymal transection is performed with Bipolar diathermy and a Da Vinci Harmonic scalpel. Hepatoduodenal ligament clamping is not utilised. Haemostatic mattress sutures are placed at the resection bed with 2/0 PDS following resection. Resected tissue is retrieved via the infraumbilical port under direct vision using an endocatch bag.

Providing there were no immediate anaesthetic or surgical complications, the nasogastric tube and urinary catheter if placed before surgery are removed before the patient is awake from general anaesthesia. Critical care unit admission is not required unless clinically indicated. Once awake, patients are allowed to eat and drink as tolerated. Pain control is achieved using local anaesthetic to the skin incisions and laparoscopically guided transverse abdominus plane block with 0.25% bupivacaine adjusted according to body weight during placement of ports. Patients are given intravenous paracetamol and oral weak opioids and/or a short course of oral non-steroidal anti-inflammatory drugs (NSAID).

#### Post-operative pathway and follow-up

Day 1: following post-resection ward round led by the team within 24 h, any intra-vascular catheters are removed. Providing the patient’s pain is adequately controlled and is mobile, patients are discharged with 28 days of prophylactic dose low molecular weight heparin (LMWH), and 10 days of oral paracetamol regularly, oral morphine or oxycodone prn, and laxative.

Clinical criteria for discharge (CCD)

1. Mobilising freely; eating and drinking adequately
2. Pain well controlled with oral analgesia
3. Independent with care for wounds/dressings
4. Self-administering tinzaparin injections
5. All lines/drains removed
6. Concerns addressed, contact details given, follow-up arranged

Day 2 and 4: clinical nurse specialist follow-up phone call, specifically enquiring about symptoms of pain, temperatures, chills, oral intake, bowel movement and adherence of LMWH. Patients are re-admitted to the acute assessment unit if there are any clinical concerns.

### Outcome measures

The primary endpoint of this study was the rate of discharge less than 24 h after surgery and secondary endpoints were to describe patient outcomes after surgical resection including rate of readmission within 30 days of discharge. Surgical complications were graded using the Clavien–Dindo classification. All statistical analysis and graphical representations were conducted and produced with R (v 4.3.2) run in R Studio (v2023.09.1+494). All mean values are reported with respective standard deviation values.

This study is a single surgeon series of cases performed between September 2022 and November 2023. Data were obtained via retrospective electronic case note review of a prospectively maintained surgical case log.

## Results

Twenty patients were included in this case series (Table 1). The mean age was 55.3 ± 15.5 years old, and the mean BMI was 26.8 ± 4.5 kg/m^2^. All except one patient were ASA grade one or two. All patients’ ECOG performance statuses were classified as zero or one.

**Table 1:** Summary of cases

Demographics, *n* = 20		%
Age (years, m ± sd)	55.3 ± 15.5	–
Female: male	11: 9	–
BMI (kg/m^2^, m ± sd)	26.8 ± 4.5	–

	*n*	%
ASA		
1	4	20
2	15	75
3	1	5
ECOG performance status		
0	5	25
1	15	75
Ethnicity		
White Caucasian	14	70
Asian, Asian British	6	30
Pre-operative interventions		
Nasogastric tube	7	35
Arterial line	11	55
Central venous catheter	9	45
Single shot spinal anaesthetic	9	45
Urinary catheter	14	70
Intro- and post-operative interventions		
Surgical drain	5	25
Intra-operative transfusion	0	0
Post-operative transfusion	1	5
Inotropic support	0	0
Patient controlled analgesia prescribed	16	80
Length of stay		
≤ 24 h	13	65
> 24 h	7	35
Reason of > 24-h admission		
Inadequate pain control	5	25
Required blood transfusion	1	5
Social reasons	1	5
Post-operative histology		
Malignant disease		
Colorectal cancer metastasis	10	50
Gallbladder fossa and hilar lymph nodes	2	10
Intra-hepatic cholangiocarcinoma	1	5
Ovarian cancer metastasis	2	10
High grade metastatic serous carcinoma	1	5
No residual malignant disease post-chemotherapy	2	10
Benign disease		
Focal nodular hyperplasia	1	5
Endometriosis lesion	1	5

**Fig. 1:**
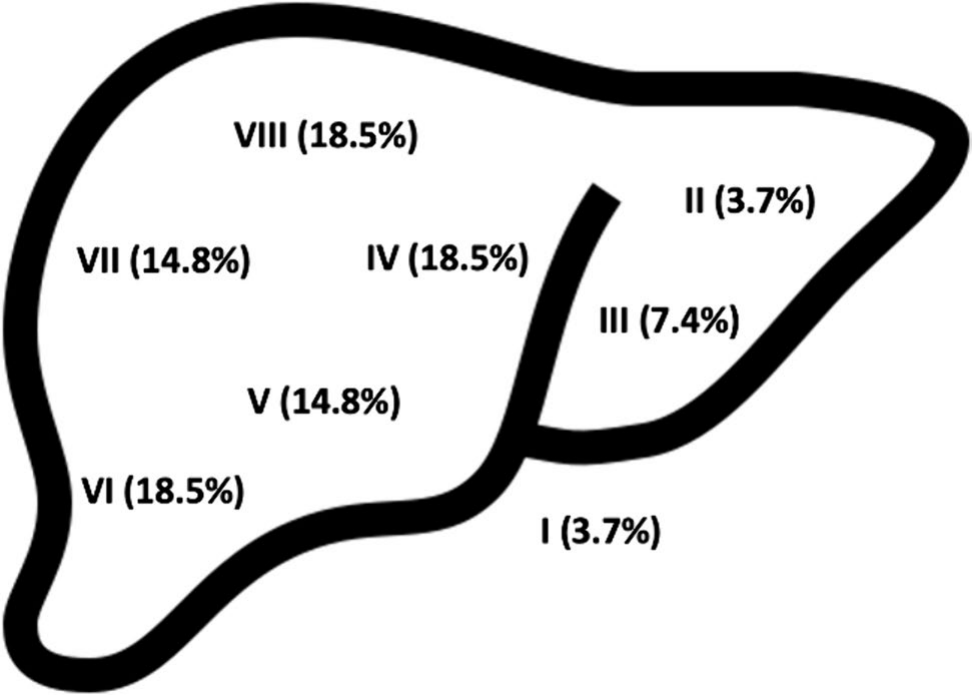
Illustration of respective liver segments and distribution (%) of where lesions were surgically resected from

Eleven patients had previously undergone major abdominal surgery and ten patients underwent neo-adjuvant chemotherapy prior to surgical resection. Fourteen cases (70%) underwent resections for solitary lesion and 6 (30%) for 2 or more lesions or other non-hepatic lesions (Table 1), the distribution and location of liver lesions excised are displayed in Fig. 1. The non-hepatic lesions included hilar lymphadenectomy (*n* = 2) and endometriosis nodule on Gerota’s fascia (*n* = 1). Perioperative and anaesthetic-related interventions are also listed in Table 1. The mean operative time was 86.6 ± 30.9 min and mean console time was 58.6 ± 24.5 min. There were no conversions to open surgery.

The maximum mean size of liver parenchyma resection was 30.7 ± 13.9 mm. Two cases (10%) were reported as R1 resections with tumour cells reported to be present microscopically at the diathermised resection margin. There was one Clavien–Dindo grade II complication; one patient required a single unit of blood transfusion. The mean pre-operative and post-operative haemoglobin was 131.7 ± 15.1 g/L and 121.0 ± 15.3 g/L, respectively.

Thirteen patients (65%) were discharged within 24 h of surgery. Of the 7 patients who remained in hospital after 24 h, five (25%) were due to inadequate pain relief and were discharged on day 2 postoperatively (< 48 h). One patient required an inpatient transfusion of red blood cells and was discharged on day 3 after repeating the blood test post-transfusion (< 72 h), and one patient could not be safely discharged within 24 h due to social circumstances and was discharged on day 3 (< 72 h).

**Fig. 2:**
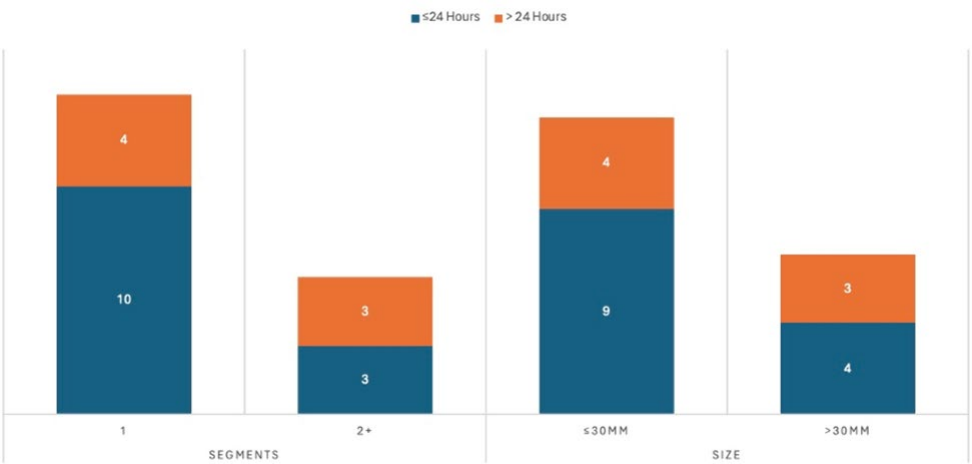
Number of patients discharged within and over 24 h when stratified against segmental lesions resected or maximum size of resection

A higher proportion of patients who underwent one segment resection or resections ≤ 30 mm were discharged within 24 h (Fig. 2). Five patients had a surgical drain inserted at the end of operation following extensive hilar dissection (*n* = 2), and tumorectomy around hepatic hilus close to vascular and biliary pedicles (*n* = 3), and they were removed prior to discharge.

When comparing patients discharged within 24 h to those discharged over 24 h (Table 2), a higher number and larger lesions were excised. A higher proportion of patients had arterial lines, central venous catheters as well as surgical drains placed. Finally, there were no Clavien–Dindo grade III or above complications, no 30-day readmission and 90-day mortality. All patients remain under active follow-up.

**Table 2:** Comparison of cases discharged within 24 h to cases discharged over 24 h

	Within 24 h	Over 24 h
*n*	13	7
Age (years, m ± sd)	59.9 ± 12.5	48.9 ± 17.9
BMI (kg/m^2^, m ± sd)	26.1 ± 3.6	28.3 ± 5.9
ECOG Performance status 0 *n* (%)	1 (7.6)	3 (42.8)
2 + segments *n* (%)	3 (24.1)	4 (42.8)
Mean size liver resection (mm, m ± sd)	28.4 ± 8.9	35.0 ± 20.5
Operative time (min, m ± sd)	78.3 ± 29.7	102.0 ± 29.1
Console time (min, m ± sd)	51.1 ± 24.8	72.6 ± 18.2
Nasogastric tube *n* (%)	4 (30.7)	3 (42.9)
Arterial line *n* (%)	7 (53.8)	5 (71.4)
Central venous catheter *n* (%)	5 (38.4)	4 (57.1)
Urinary catheter *n* (%)	10 (76.9)	5 (71.4)
Surgical drain *n* (%)	2 (15.3)	3 (42.9)
Single shot spinal anaesthetic *n* (%)	5 (38.4)	4 (57.1)
Patient controlled analgesia *n* (%)	8 (61.5)	7 (100)
Malignant disease *n* (%)	12 (92.3)	6 (85.7)

## Discussion

Robotic minor liver resections have previously been demonstrated in other series to be safe and led to a shorter length of stay when compared to open surgery [12, 13]. Similarly, day-case laparoscopic minor liver resections have also been demonstrated to be feasible in the appropriately selected patient [16]. This is the 1st series of robotic day-case liver resection in the UK based on our well-established robotic hepatobiliary and pancreatic programme. Our results showed that the day-case liver resection is achievable and safe for patients to be discharged home within 24 h of liver resection.

The primary end point of discharge within 24 h of robotic liver surgery was achieved in 65% of patients following the introduction of robotic day-case liver resection in our series. There were no 30-day re-admissions. The main reason for patients not discharged within 24 h was inadequate pain control requiring further inpatient pain management. One patient required a blood transfusion following surgery but had a lower pre-operative haemoglobin count of 105 g/L. The results highlighted the need for preoperative optimisation of patient’s baseline physiological parameters prior to major abdominal surgery and effective approach for pain control postoperatively.

Inadequate pain control was the dominant cause of discharges beyond 24 h. Our current combination of local anaesthetic to the skin incisions, laparoscopic-guided transverse abdominus plane block (TAP), intra-venous paracetamol, and oral weak opioids and/or a short course of NSAIDs is based on our experience of other day-case laparoscopic general surgical procedures such as cholecystectomy and inguinal hernia repair which may need to be optimized to improve the effectiveness of postoperative pain control. Laparoscopic guided TAP blocks have been shown to be an effective mode of pain relief following minimally invasive pancreatectomy [17], bariatric surgery [18] and open large ventral hernia repairs [19].

Although not part of the pathway, immediately postoperatively 80% of patients were prescribed an opioid-based intra-venous patient-controlled analgesia (PCA) pump which is extrapolated from our major liver or pancreatic resection pathways. No information was available on the amount of usage of the PCA. Patients discharged over 24 h had more lesions and larger size of liver parenchyma resected (Table 2). The higher proportion of arterial line, central venous catheter and surgical drain placement also reflects the higher complexity of the operation. Other measures to improve post-operative pain could also be considered as part of a pathway particularly for cases that involve resection more than one lesion. Options may include the application of intra-peritoneal local anaesthetic, which has been shown to reduce pain and opioid consumption following major laparoscopic abdominal resection surgery [20, 21].

When considering the baseline demographics of this cohort, the mean BMI was 26.8 ± 4.5 kg/m^2^, ASA grade 2 75% and performance status one 75%. There were no significant Clavien–Dindo grade III or above morbidities, readmissions within 30 days of discharge nor 90-day mortality demonstrating the feasibility and safety of robotic day-case liver resection. With shorter length of stay and enhanced recovery, not only are there cost-saving benefits, but reduced length of stay has a positive impact on the patient’s physical recovery and mental well-being in the post-operative period [22].

Half the study cohort had also undergone previous major abdominal surgery, but this did not affect the robotic completion rate, with no conversions to open surgery in this case series. It is the authors’ experience that as long as a pneumoperitoneum can be introduced safely, it is safe and possible to perform extensive adhesiolysis using the robotic platform to create intra-abdominal space for robotic surgery. The authors believe the mean operative time of 86 min and mean console time of 58 min are not inferior to our own experience with open non-anatomical liver resections. Direct comparison with published literature is difficult as most series report an average operating time for combined minor and major robotic liver resections of approximately 4 h [23–25]. Our series represented very well a range of hepatic tumours normally requiring minor liver resection, and the result confirmed that this could be achieved safely via robotic approach with satisfactory oncological outcomes for cancer cases judged by the R0 status and minimal blood loss as evidenced by a stable post-operative haemoglobin level.

It is also important to consider the patient’s expectations and perspective. As reported, a significant portion of patients have undergone previous cancer-related treatments, it is important to consider the burden of such diagnoses and that patients don’t feel they are pushed out of the hospital or abandoned following surgical intervention. In our experience, the scheduled phone calls following discharge help to give patients peace of mind but also act as an important safety net to identify any potential complications to allow for intervention promptly. Patient’s expectations of pain management are also important, similar to day-case laparoscopic cholecystectomy, patients should be counselled that while not pain-free, pain control is achieved with a multimodal approach and that being pain-free before discharge is not a realistic expectation.

The main limitation of this study is that it is a relatively small cohort of a very selected patients. The procedures are performed in a unit which performs high-volume robotic hepatobiliary and pancreatic surgery so the results may not be immediately generalisable and is susceptible to selection bias. The authors would suggest centres should be routinely performing major robotic liver resections before embarking on a similar program. Nonetheless, it sets a benchmark for minor liver resection and robotic liver resection.

This study focused on the immediate and peri-operative outcomes only. Long term clinical and oncological outcomes should be compared to open or laparoscopic data to ensure there is no inferiority. From a patient’s perspective, quality of life and patient experience measures can be used to assess the impact of this pathway on patients. Cost-related analysis may also be useful when assessing the health economic-associated benefits and may help decide whether this approach should be adopted in a publicly funded health system.

## Conclusion

This case series demonstrates that robotic day-case liver resection is safe and feasible in the appropriately selected patient. A multimodal approach to pain relief is required to allow for patients to be discharged comfortably. Robust follow-up pathways must be in place to allow for the safe implementation of this approach, to monitor for any complications and to allow intervention as required in a timely manner.

## Data Availability

The data, methods used in the analysis, and materials used to conduct the research will be made available to any researcher for the purposes of reproducing the results of replicating the procedure.
